# Pelvic Flexure Enterotomy in Horses: Scoping Review and Synthesis of the Literature (1996–2026)

**DOI:** 10.3390/vetsci13090930

**Published:** 2026-09-09

**Authors:** Valeria Albanese, Paola Straticò, Amelia Munsterman

**Affiliations:** 1 Tierärztliches Kompetenzzentrum für Pferde Großwallstadt Altano GmbH, Niedernberger Str.9, 63868 Großwallstadt, Germany; vzalbanese@gmail.com; 2 Department of Veterinary Medicine, Università Degli Studi di Teramo, Piano D’Accio, 64100 Teramo, Italy; 3 Department of Large Animal Clinical Sciences, Michigan State University College of Veterinary Medicine, 736 Wilson Road, East Lansing, MI 48824, USA; munster4@msu.edu

**Keywords:** equine, pelvic flexure enterotomy, suture technique, scoping review, surgery

## Abstract

This scoping review maps the available literature aimed at identifying the most appropriate technique for suturing pelvic flexure enterotomies in horses. A comprehensive search of Web of Science, Google Scholar, and the National Center for Biotechnology Information was conducted for studies published between January 1996 and February 2026, using terms related to pelvic flexure enterotomy and equine patients. From 22,146 records, only five ex vivo studies on healthy equine colon specimens met the inclusion criteria. The studies compared variables such as suturing time, suture pattern, material, bursting pressure, luminal diameter reduction, and cost. Double-layer hand-sewn closure generally achieved the highest bursting pressures, although it required longer operative time, while TA90 stapling was the most expensive option. Because ex vivo models cannot reproduce tissue healing or host–construct interactions, the clinical applicability of the available evidence remains limited. Nonetheless, a full-thickness simple continuous pattern oversewn with a continuous Cushing pattern appears to offer the most dependable mechanical security until stronger in vivo evidence becomes available.

## Simple Summary

Pelvic flexure enterotomy is a common surgical procedure used in horses with large colon impactions, but there is no clear agreement on the best way to close the intestinal incision afterward. This review examined the available scientific literature to compare different closure techniques, including hand-sewn single- and double-layer patterns and stapling devices. Although more than 22,000 records were initially identified, only five studies met the inclusion criteria, and all were performed ex vivo, meaning on healthy colon tissue outside the living animal. Therefore, these studies can measure mechanical outcomes, such as how much pressure a closure can resist before leaking or bursting, but they cannot show how the tissue heals after surgery or how complications may develop in clinical cases. Overall, double-layer hand-sewn closure, using a full-thickness simple continuous pattern oversewn by a continuous Cushing pattern, appeared to provide the strongest mechanical seal. However, this technique took longer to perform than some alternatives. Stapling with TA90 was faster but more expensive. Based on current evidence, surgeons should choose the closure method by balancing mechanical security, surgical time, tissue condition, and cost, while recognizing that clinical studies are still needed.

## 1. Introduction

Pelvic flexure enterotomy provides direct access to the large-colon lumen, allowing removal of luminal contents and gross assessment of the mucosa. It is most frequently used when firm or voluminous impactions cannot be adequately resolved by medical treatment. Such impactions may consist of dehydrated or poorly masticated feed material, but sand or other indigestible foreign material may also be present [1,2,3]. Decompression of the large colon through the pelvic flexure can additionally be useful when small-colon disease is present, because reducing the amount of ingesta entering an inflamed or compromised distal bowel may limit further distension or recurrence. In horses with concurrent cecal and large-colon impaction, large-colon evacuation may facilitate movement of cecal contents. Enteroliths or fecaliths that can be manipulated to the pelvic flexure can likewise be removed through this approach [1,3,4]. Pelvic flexure enterotomy has also been proposed during small-intestinal surgery because large-colon decompression may be associated with a lower occurrence of postoperative reflux [5].

Despite being routinely performed, pelvic flexure enterotomy adds potential morbidity to abdominal surgery. It prolongs the procedure and requires additional intestinal manipulation, while opening the colon changes the operative field from clean to clean-contaminated [6]. Depending on the closure construct, suture material may remain exposed on the serosal surface and contribute to local inflammation or adhesion formation [7,8,9,10]. Hemorrhage from the enterotomy site is another recognized complication [11].

The objectives of this scoping review are to identify, evaluate, and summarize the findings of all relevant individual studies describing techniques for pelvic flexure enterotomy closure in horses, to identify knowledge gaps in the literature, and, if possible, to identify the most suitable technique for its closure based on the current, relevant literature.

## 2. Materials and Methods

Peer-reviewed articles providing detailed data about techniques for performing and closing pelvic flexure enterotomies in horses, published from January 1996 to February 2026, were included in the study. The review was conducted and reported following the PRISMA 2020 guidelines [12]. Inclusion criteria were defined according to the PICO principles (See Supplementary file):

- Population: Horse/specimen that received a pelvic flexure enterotomy;
- Intervention: Pelvic flexure enterotomy and closure;
- Comparison: Between different techniques and suture materials;
- Outcome: A total of 3 out of 4 of the following measurable variables must be quantified:
  ○ Closure time;
  ○ Bursting pressure;
  ○ Diameter reduction;
  ○ Suture material.

Ex vivo and in vivo studies were included. To be included, studies had to be published in English.

Non-English language publications, abstracts, scientific proceedings, doctoral dissertations, citations, and book chapters were excluded. Studies not specifically evaluating pelvic flexure enterotomy techniques were excluded.

A comprehensive literature search was conducted through three online databases: Web of Science (WOS), Google Scholar, and the National Center for Biotechnology Information (NCBI) from January 1996 up to February 2026. Reference lists of the included studies and relevant reviews were hand-searched to identify additional studies. This scoping review was not registered.

The search strategy was designed to capture all relevant studies using a combination of keywords and MeSH terms related to “pelvic flexure enterotomy” and “horse” or “equine.” The exact search strings for each database were (Table 1):

- NCBI: (“Horses”[Mesh] OR horse*[tiab] OR equine[tiab])AND(enterotom*[tiab])AND(“large colon”[tiab] OR “ascending colon”[tiab] OR “pelvic flexure”[tiab]).
- Google Scholar: (horse OR equine) AND enterotomy AND (“pelvic flexure” OR “large colon” OR “ascending colon”).
- WOS: horse* (Topic) or equine (Topic) and enterotomy* (Topic) and pelvic flexure (Topic) and suture (Topic) or closure (Title) or suturing (Author).

Two authors independently screened the titles and abstracts of the retrieved records for eligibility. Full texts of potentially eligible studies were then independently assessed. Disagreements were resolved through discussion or, if necessary, by consulting a third author.

Data were extracted independently by two reviewers using a standardized data extraction form. Extracted information included study characteristics (authors, year, and study design), population details, description of the enterotomy techniques, closure methods, and outcomes. Discrepancies were resolved through discussion or consultation with a third reviewer.

Given the diversity in study designs, enterotomy closure techniques, suture materials, and reported outcomes, a narrative synthesis was deemed the most appropriate method for data synthesis. The narrative synthesis focused on categorizing studies by the type of closure technique (e.g., full-thickness continuous Cushing or seromuscular Cushing), suture material (e.g., polyglactin size 2-0), and types of specimens (client horses to slaughter horses) used. This approach allowed for a comprehensive examination of the evidence while accounting for the variability across studies. A comparative analysis was conducted to explore the differences in average closure time, bursting pressure and diameter reduction between various suture materials and techniques. Using descriptive statistics to summarize the findings, a comparative overview of available literature was provided. Due to the narrative nature of the synthesis and the heterogeneity among studies, statistical pooling of the results was not performed.

As a scoping review, this study did not involve primary data collection, and ethical approval was not required. All the analyses were based on previously published data.

## 3. Results

**Figure 1:**
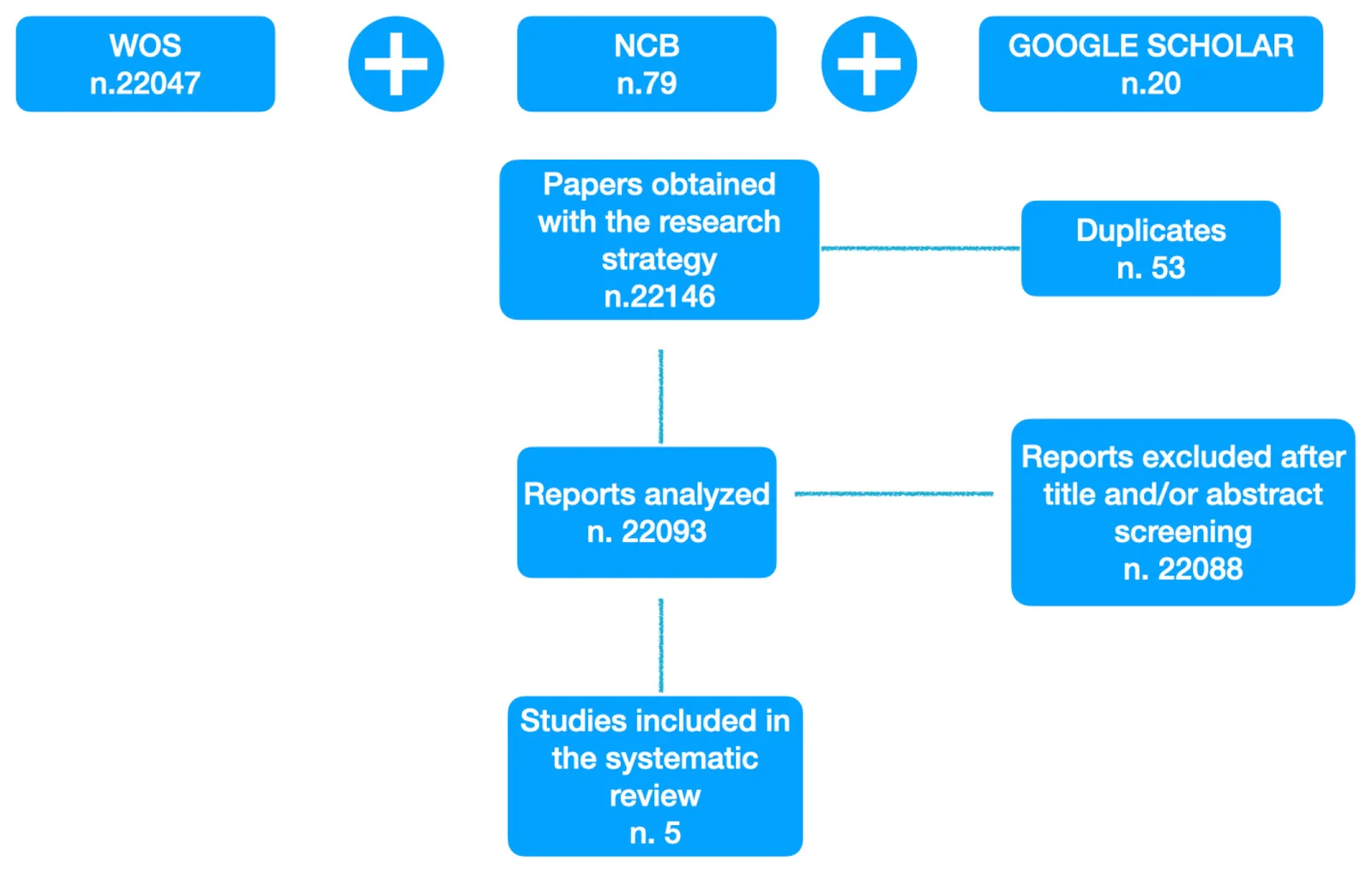
Flowchart showing the methods used for the systematic study (WOS: Web of Science; NCBI: National Center for Biotechnology Information; Google Scholar). Respectively 22,047, 79 and 20 papers were obtained from each database; of these 53 were removed as duplicates. Twenty-Two-thousand and ninety-three papers underwent a title and/or abstract assessment at the end of which 5 were included for analysis.

The flowchart (Figure 1) shows the results of the systematic analysis. The research strategy yielded the following results: WOS 22,047 papers, NCBI 79 papers, Google Scholar 20 papers. After removal of duplicates and abstract assessment, a total of five studies were included in the analysis. This review encompassed five studies conducted from 2012 to 2020, assessing techniques and materials in pelvic flexure enterotomy in horses. These studies varied in their approach, describing a range of enterotomy closure techniques, suture materials, and types of specimens.

Details about the studies that were included in the scoping review are summarized in Table 2.

No in vivo studies meeting our inclusion criteria were found. The flowchart describing the methodology of study selection is shown in Figure 1.

Rosser et al. (2012) [13] compared a full-thickness simple continuous pattern oversewn with a Cushing pattern (polydioxanone size 2-0) with a single-line staple closure (Thoraco-Abdominal-TA90 surgical staple, average length: 6.0 cm) on 13 client horses that were euthanized for reasons not related to the gastrointestinal tract (respectively seven and six). The study reported on time and bursting strength using a fluid pump and diameter reduction.

Gandini et al. (2013) [14] investigated four different closure techniques in 48 fresh intestinal tract specimens collected from slaughtered horses, divided into two main groups, according to the length of the pelvic flexure enterotomy (5 cm vs. 10 cm). Each group was divided into four subgroups: single-layer Cushing seromuscular suture pattern, double-layer with a simple continuous full-thickness layer oversewn with a Cushing seromuscular suture, both with 2-0 polyglactin 910, TA90 surgical staple, and stainless-steel staple. Their study reported on time and bursting strength after air filling measured with a modified gas inflation tank test and diameter reduction.

Aldrich et al. (2017) [10] analyzed a full-thickness simple continuous suture pattern incorporating the seromuscular layer, a full-thickness simple continuous pattern followed by a Cushing pattern without severing the suture, and double-layer closure with suture line reversal with a simple continuous pattern oversewn with a Cushing pattern on 18 client horses (6 horses each pattern), euthanized for reasons other than abdominal disease. Tests were performed within 4 h of euthanasia. In the study by Aldrich et al. (2017) [10] a 2-0 Glycomer 631 material was utilized (average length: 10.0 cm). Data were provided on time and bursting strength after filling with tap water through an infusion pump and on diameter reduction.

Giusto et al. (2019) [15] focused on a hand-sewn double-layer closure technique that included a full-thickness single continuous suture pattern oversewn with a Cushing pattern. Eight cm pelvic flexure enterotomies were closed within 6 h of collection in 24 slaughter horses, allocated to two groups. In one group a barbed glycomer 631 (V-Loc 90) was employed. In the other group an unbarbed glycomer 631 size 2-0 was employed. The study reported on time, bursting pressure and cost.

Sinovich et al. (2020) [16] compared four different sutures on a 10 cm enterotomy created over 24 fresh large colon specimens, collected 6 h before testing: a single layer continuous Cushing absorbable pattern, a double layer pattern (seromuscular oversewn with Cushing) with absorbable suture material (polysorb Size 2-0), a single layer unidirectional barbed suture pattern and a bidirectional barbed one (V-Loc 90 wound closure device), and reported on time and bursting pressure using a modified gas inflation water tank test, diameter reduction, and cost.

Comparison of the outcomes described for each paper is summarized in Table 2.

Length of enterotomy also varied, although it was standardized within each study. In three out of five studies, it was 10 cm [10,14,16]; in one study, 8 cm [15]; and in one, 6 cm [13]. In one paper two different lengths were tested for the same variables [12].

Time to perform the suture, suture pattern and bursting pressure were evaluated in all cases. In all the studies, hand-sewn double-layer closures showed a significantly longer time to perform compared to other techniques. Bursting pressures were not significantly different between double- and single-layer hand-sewn closures in all but one study [14] where the double-layer hand-sewn closure demonstrated the highest bursting pressure amongst all the studies. Hand-sewn single-layer closures, performed both with barbed and unbarbed material, were faster to perform when compared to hand-sewn double-layer closures.

Information about diameter reduction was available in four out of five studies. Of these, three were expressed as a percentage of reduction [13,15,16] and one as diameter in mm and lumen diameter ratio [10]. The results between studies are not consistent, showing the least diameter reduction for TA-90 closures compared to hand-sewn techniques in two studies [9,11], while the larger reduction was obtained after closures performed with barbed sutures [16]. No significant differences among techniques were found according to Gandini et al. (2013) [14] and Aldrich et al. (2017) [10].

Concerning the cost of the procedure, the TA90 closure was the most expensive, followed by the hand-sewn double-layer closures. Comparison of cost between barbed and unbarbed suture material was available only in one study [16], without direct comparison of the data.

## 4. Discussion

This scoping review maps the evidence available for closure of equine pelvic flexure enterotomies and compares the outcomes most consistently reported across experimental studies: closure time, bursting pressure, change in luminal diameter, and cost. Although pelvic flexure closure has been investigated experimentally [10,14,16], in vivo [17], and in clinical series [18,19], the literature does not currently support a definitive evidence-based choice of one technique for routine clinical use.

The search was restricted to the preceding 30 years and, after application of the predefined eligibility criteria, only five studies qualified for inclusion; all five were ex vivo investigations. Other publications from this period describe pelvic flexure enterotomy in horses undergoing emergency laparotomy [20,21,22] but generally evaluate the procedure as one component of a broader surgical intervention rather than as an isolated technique. Clinical outcome in those studies is influenced by numerous variables, including the primary lesion, tissue perfusion, cardiovascular and metabolic status, and surgeon-related factors [23]. They also did not provide the combination of closure-specific outcomes required by the present review and were therefore not eligible for inclusion.

The experimental setting of ex vivo studies facilitates controlled comparison of closure constructs and their immediate mechanical properties. Conversely, these models cannot reproduce perfusion, inflammation, healing, contamination, postoperative loading, or other patient-specific biological responses that may alter construct performance after surgery.

Pelvic flexure enterotomy is performed within the complex setting of equine emergency laparotomy, in which postoperative outcome depends on multiple disease- and patient-related factors. Emptying the large colon can facilitate decompression, reduce luminal contents, and improve manipulation and repositioning of the bowel. These benefits must be weighed against additional operative time and the consequences of creating and closing a contaminated intestinal incision. Accordingly, an optimal closure would combine adequate mechanical security with rapid application and minimal narrowing of the lumen, without introducing clinically important adverse effects.

Among the constructs evaluated, the conventional hand-sewn double-layer closure is the most commonly used method and is recognized as the most reliable [24]. It offers strong mechanical performance and is relatively inexpensive in terms of suture material, but consistently requires more time to complete [10,13,14,16]. Whether the additional minutes required for closure materially affect outcome after colic surgery remains uncertain and may be clinically minor [10]. Across the ex vivo literature, double-layer constructs tolerated pressures exceeding reported in vivo large-colon luminal pressures, which may reach approximately 58 mmHg [25,26]. Leakage was not observed during testing in the included studies. The effect on luminal caliber was more variable, with reported reductions ranging from 2.07% to 14.18% depending on technique and measurement method [10,13,14,17]. In principle, narrowing at the pelvic flexure could be most relevant early after surgery, when inflammation and impaired motility may already compromise transit [10,15,27], and could impede passage of ingesta once feeding is resumed [14,27].

The clinical importance of this experimentally measured luminal narrowing remains poorly established and is likely to be greatest in animals with a small colonic diameter, such as foals or miniature horses. Evidence from an earlier in vivo experiment provides some biological context: a double-layer closure produced satisfactory apposition of the colonic wall and mucosal healing and performed better histologically than the single-layer Utrecht and three-layer constructs evaluated [18]. That study was not included in the present scoping review because its outcomes were based on healing, microangiography, and histology rather than the prespecified mechanical variables of closure time, bursting pressure, and diameter reduction.

A modification of the two-layer technique allows the same suture strand to be continued into the Cushing layer by reversing direction at the terminal knot of the initial full-thickness simple continuous layer. In the study evaluating this configuration, its resistance to bursting was comparable with that of the conventional two-layer construct, but the modification did not provide a meaningful reduction in closure time [10].

Survey data indicate that double-inverting patterns are used by a notable proportion of equine surgeons [28] since their first description in 1982 by Huskamp et al. [29] despite the absence of ex vivo biomechanical testing of these constructs. The limited clinical literature includes postoperative hemorrhage associated with enterotomy closure [11], raising concern that an entirely inverting construct may not reliably incorporate or compress submucosal vessels. Experience from other gastrointestinal and reproductive procedures supports deliberate inclusion of the submucosa when hemostasis is important: canine gastrotomy is commonly closed with a full-thickness continuous layer, with or without an additional inverting layer [30,31], and two-layer closure incorporating mucosa and submucosa has been recommended for equine hysterotomy to limit postoperative hemorrhage [32]. On the evidence currently available, a double-inverting pattern cannot be recommended for pelvic flexure enterotomy closure.

Single-layer inverting Cushing constructs also withstood pressures above the in vivo colonic values reported in the literature (up to approximately 58 mmHg) [10,14,17]. Their bursting pressures were comparable with or below those of double-layer hand-sewn constructs in the available comparisons [10,14,17], while closure was consistently faster. Differences in luminal narrowing were not statistically significant between several tested constructs. Mechanical performance alone, however, does not establish biological suitability. In the in vivo study by Young et al. [7], a single-layer Utrecht closure was associated with postoperative colic, leakage, and adhesion formation. Histologic assessment showed extensive serosa-to-serosa apposition but inadequate alignment of the muscularis and submucosa and a persistent mucosal gap, findings that could interfere with normal tissue healing.

Clinical use of the TA-90 stapling device has been described in substantial case series. Ellis et al. [18] reported its application in more than 200 horses without identified technique-related complications and noted a markedly shorter closure time than with a conventional two-layer hand-sewn technique (approximately 3 versus 15 min), although the staple line was not oversewn. Rosser et al. [19] subsequently described 84 clinical cases with outcomes and complication rates comparable to those expected after large-colon surgery. In the ex vivo comparison by Rosser et al. [13], TA-90 closure achieved bursting pressures like the hand-sewn double-layer construct while producing less luminal narrowing and requiring less time.

The apparent time advantage of TA-90 closure was not consistent across experimental designs. Gandini et al. [14] found a clear benefit for 5 cm enterotomies, which could be closed with a single cartridge; for longer incisions requiring additional stapling, total closure time approached that of hand-sewn techniques. In the same study, TA-90 constructs had lower bursting pressures than double-layer hand-sewn closures, although the values remained above reported physiologic intraluminal pressures. Reduction in luminal diameter was not significantly different among the compared techniques.

Skin staples represent a simpler mechanical alternative but have very limited supporting evidence. In the ex vivo study by Gandini et al. [14], skin-stapled closures were rapid, technically straightforward, and inexpensive. Their bursting pressure was lower than that of the conventional hand-sewn double-layer closure but remained above published in vivo colonic pressures, and luminal narrowing was like that produced by the other tested constructs. No in vivo experimental study or clinical outcome study has yet validated this method for pelvic flexure enterotomy closure.

## 5. Conclusions

The available evidence does not suffice for recommending a single closure technique as the most appropriate option for clinical use. The current findings indicate that the traditional double-layer hand-sewn closure may provide favorable biomechanical characteristics, including greater resistance to leakage and mechanical failure under controlled experimental conditions. However, these conclusions are derived exclusively from ex vivo studies, which assess the immediate mechanical behavior of tissues and sutures but cannot reproduce the complex biological and physiological processes that occur in vivo. Factors such as tissue perfusion, inflammation, edema, healing response, suture degradation, contamination, postoperative loading, and patient-specific variability may substantially influence the performance of a closure technique in clinical practice. Furthermore, differences in experimental protocols, tissue models, operator technique, suture materials, and outcome definitions limit direct comparison among studies and reduce the generalizability of the reported results. Well-designed in vivo investigations and comparative clinical studies, controlling for relevant clinical variables and specifically evaluating pelvic flexure closure techniques and clinically relevant outcomes, including leakage, complications, healing, operative time, and long-term function, are required before definitive recommendations can be made.

## Figures and Tables

**Table 1:** Summary of the research strategy. The databases that were used (“Databases”), the search terms, the hits from each database and the order in which sites were searched are shown.

Database	Search Terms	Results	Search Chronology
National Library of Medicine	https://pubmed.ncbi.nlm.nih.gov/?term=(%22Horses%22%5BMesh%5D%20OR%20horse*%5Btiab%5D%20OR%20equine%5Btiab%5D)%20AND%20(enterotom*%5Btiab%5D)%20AND%20(%22large%20colon%22%5Btiab%5D%20OR%20colon%5Btiab%5D)&filter=lang.english&filter=other.excludepreprints&filter=years.1996-2026	20	1st (19 February 2026)
Web of Science	https://www.webofscience.com/wos/woscc/summary/330f67fd-98ea-423b-b604-d8defefdf28b-019f819e25/relevance/1	22.047	2nd (19 February 2026)
Google Scholar	https://scholar.google.com.hk/scholar?hl=zh-TW&as_sdt=0,5&q=q%3D(horse%2BOR%2Bequine)%2BAND%2Benterotomy%2BAND%2B(%2522+pelvic%2Bflexure%2522%2BOR%2522+large%2Bcolon%2522%2BOR%2522+ascending%2Bcolon%2522)	79	3rd (19 February 2026)

**Table 2:** Papers included in the scoping review, suture material and suture technique, number of specimens used and measured outcome. Within a column, means with the same superscript are not statistically different (TA: thoracoabdominal; HS2: hand-sewn 1 layer; HS1: hand-sewn 1 layer; S-L: single layer; D-L: double layer; NE: not evaluated; USD: United States Dollar).

Author(s)	n. Specimen	Length of Enterotomy	Suture Technique and Material	Measured Outcome			
				Time (min)	Bursting Pressure	Diameter Reduction	Cost
Rosser et al. 2012 [13]	13	6 cm	TA90 stapling device	3.31 ± 0.85 ^a^	116.7 ± 5.2 cm H_2_O	4.7 ± 2.8% ^a^	NE
			Hand-sewn double-layer polydioxanone	15.8 ± 1.41 ^b^	111.1 ± 16.9 cm H_2_O	11.98 ± 2.2% ^b^	NE
Gandini et al. 2013 [14]	48	10 cm	Hand-sewn double-layer polyglactin 910 (HS2)	12.00 ± 0.71 ^a^	182.86 ± 14.5 ^a^ mmHg	8.46 ± 3.75%	110.44 euro
			Hand-sewn single-layer polyglactin 910 (HS1)	6.31 ± 0.43 ^b^	115 ± 16.31 ^b^ mmHg	8.29 ± 4.15%	5.22 euro
			Skin staples (SKS)	6.15 ± 0.62 ^b^	95.71 ± 8.36 ^b^ mmHg	10.35 ± 4.01%	9.35 euro
			TA90 stapling device (TA90)	9.77 ± 0.21 ^a^	69.21 ± 15.01 ^b^ mmHg	14.69 ± 5.15%	198–396 euro
Aldrich et al. 2017 [10]	18	10 cm	Hand-sewn single-layer	5.27(1.5–9.07) ^a^	93.4 (70.3–116.4) mmHg	97 mm	NE
			Hand-sewn double-layer	8.72 (7.75–9.7) ^b^	88.8 (78.4–99.2) mmHg	107 mm	NE
			Hand-sewn double-layer reversing at the knot	8.42 (7.12–9.73) ^b^	92.9 (79.8–106) mmHg	107 mm	NE
Giusto et al. 2019 [15]	24	8 cm	Hand-sewn double-layer glycomer 631	12.38 ^a^	183.3 mmHg	NE	15.1 euro
			Hand-sewn double-layer barbed glycomer 631	11.65 ^b^	188.9 mmHg	NE	84.12 euro
Sinovich et al. 2020 [16]	24	10 cm	Hand-sewn single-layer absorbable (S-L)	5.0 ± 0.18 ^a^	117.6 ± 11.69 ^a^ mmHg	3.9 ± 2.93% ^a^	4.48 USD
			Hand-sewn double-layer absorbable (D-L)	8.9 ± 0.47 ^b^	178.5 ± 9.79 ^a^ mmHg	5.4 ± 3.33% ^b^	4.48 USD
			Hand-sewn single-layer unidirectional barbed (V-Loc)	4.5 ± 0.55 ^a^	91.6 ± 5.57 ^b^ mmHg	11.6 ± 4.16% ^c^	43.58 USD
			Hand-sewn single-layer bidirectional barbed (Quill)	4.3 ± 0.4 ^a^	87.5 ± 8.69 ^a^ mmHg	3.2 ± 3.06% ^c^	32.09 USD

## Data Availability

No new data were created or analyzed in this study. Data sharing is not applicable to this article.

## Supplementary Materials

The following supporting information can be downloaded at: https://www.mdpi.com/article/10.3390/vetsci13090930/s1. Supplementary file: PRISMA 2020 Checklist.
